# Supplementary material for: Are carbon emissions trading and green financial instruments synergistic? -Comprehensive quantitative research based on content analysis
Source: PLoS One. 2024 Mar 7;19(3):e0298601. doi: 10.1371/journal.pone.0298601 (PMC10919601; doi:10.1371/journal.pone.0298601)
Supplement: S1 Data — (ZIP) [file pone.0298601.s001.zip › Data/地级市Z_F工作报告.zip!/╕ú╜¿/╚2├≈╩╨/└┤╫╘╣1⁄2╓┌║┼í╛┬φ┐╦╩2╛▌═°í┐_2010.docx]

各位代表：

　　现在，我代表三明市人民政府向大会报告政府工作，请予审议，并请市政协委员和其他列席人员提出意见。

　　一、2009年工作回顾

　　过去的一年，我市各级政府在省委、省政府和市委的正确领导下，认真贯彻党的十七大、十七届三中、四中全会精神，深入学习实践科学发展观，全面落实国务院《关于支持福建省加快建设海峡西岸经济区的若干意见》及省《实施意见》，有效应对国际金融危机，全力保增长、保民生、保稳定，经过全市人民共同努力，经济呈现回升向好的态势，各项事业持续发展，较好地完成了市十一届人大四次会议确定的目标任务。初步统计，全市生产总值734.24亿元，增长13.4%；三次产业结构从上年的20.8:47.8:31.4调整为20:48.4:31.6；全社会固定资产投资678.26亿元，增长32.3%；地方级财政一般预算收入37.96亿元，增长15.2%；按可比口径实际利用外商直接投资 2.61亿美元，增长10.1%；外贸出口7.62亿美元，增长8.9%；社会消费品零售总额217.36亿元，增长17.4%；城镇居民人均可支配收入16500元，实际增长11.1%；农民人均纯收入6327元，实际增长9.6%；居民消费价格指数98.5%；城镇登记失业率3.6%；人口自然增长率5.8‰；预计单位生产总值能耗下降6%左右；二氧化硫削减率为5.5%，化学需氧量削减率为0.2%。一些重要领域、重点项目取得突破：

　　——泉（州）三（明）高速公路全线通车；永（安）武（平）高速公路三明段基本完工，永（安）宁（化）高速公路全线施工，建（宁）泰（宁）高速公路动工建设；向（塘）莆（田）铁路三明北站动工；三明“无水港”项目开工建设。

　　——机械及汽车产业、纺织产业产值分别突破100亿元；新增5枚中国驰名商标。

　　——永安市成为全国林业改革与发展示范区；清流台湾农民创业园升格为国家级台湾农民创业园；尤溪县被评为中国革基布名城。

　　——三明市和永安市、泰宁县被评为全国科技进步先进市（县），9个县（区）通过国家科技进步考核。

　　——我市被确定为创建国家级创业型城市、全国第二批再生资源回收体系建设试点城市、全国商务综合执法试点城市。

　　这一年，我们的工作与成效主要体现在以下四个方面：

　　（一）应对总体有效

　　面对国际金融危机，迅速开展政策研究，落实中央和省应对危机、扩大内需的政策措施，出台扩大投资、激活消费、扶持企业、保障资金等一系列举措，强化经济运行的综合调度和协调服务，帮助企业增强信心、搞好生产、开拓市场，促进经济增长逐月回升。

　　用足国家投资政策。增加投资强度，加快项目进度，投资对经济增长的贡献率达到68.9%。围绕重点建设，做到重点保障、重点服务、重点突破，150个省、市级重点项目完成投资163.86亿元，63个项目建成投产。围绕基础设施建设，超常规策划、争取、推进，制定实施《三明市公路交通发展行动计划》，完成三明中心城市快速通道、三明至明溪联络线和尤溪至莆田兴化湾、厦门至沙县、永安至漳州高速公路可研报批，省道改造开工里程达到365公里，建成农村公路750公里；南三龙铁路预可研报告通过审查，浦建龙梅铁路完成预可研设计方案评选工作，长永泉铁路前期工作稳步推进；三明沙县机场优化场址通过国家民航局审查，可研报告上报国家发改委，进场公路动工建设；永安火电厂扩改等项目加快实施，500千伏高压电网实现“南联北接”，地区电网输送能力提高。围绕生产性项目建设，加快建设投资亿元以上项目特别是63个在建工业项目，完成投资52.54亿元，增长55.8%。扎实推进重大项目前期工作，三明示范快堆核电项目取得明显进展。

　　用活适度宽松的货币政策。实行政府支持金融、金融刺激经济的办法，推行金融机构服务中小企业的7项承诺，金融机构新增贷款151.63亿元，比上年增加1.4倍，其中中小企业新增贷款81.07亿元，增长28%。拓宽融资渠道，争取地方政府债券转贷资金3.23亿元，新增开发性金融资金34.04亿元，30家担保机构提供贷款担保58.72亿元，增长51.1%。鼓励永安智胜公司进入证监会首次发行股票审核程序；推进村镇银行发展，筹建小额贷款公司、三明城区农村商业银行，融资难问题得到缓解。

　　用好积极的财政政策。落实国家扶持政策，主动争取支持，获得国家、省补助资金46.04亿元，增长27%。其中，强农惠农资金6.47亿元，民生水利基础设施建设资金2.6亿元。围绕国家新增投资导向，迅速策划、上报、对接项目，自2008年第四季度以来，共争取291个中央新增投资项目，获得中央预算内投资5.43亿元，建成168个项目。取消和停征100项行政事业性收费，落实增值税转型改革政策，为企业和社会减负7.33亿元。

　　（二）落实总体有力

　　围绕海西发展战略抓落实。着力先行先试，制定贯彻落实国家、省加快建设海西部署的实施意见，金属材料及深加工等产业列为海西重点发展产业,装备制造业等基地列为海西重点建设基地,三明生物医药等产业基地列入海西高新技术产业基地,海西三明台商投资区、海西三明现代物流产业开发区的开发建设得到省上支持。深化两岸合作，成功举办第五届海峡两岸林业博览会暨投资贸易洽谈会,重大项目签约数量和投资规模超过上届；加快海峡两岸现代林业合作实验区和清流台湾农民创业园建设，规划建设海峡两岸林业博览园、永安闽台现代竹业合作示范园；举办首届闽台妇女经贸论坛,办好第十五届世界客属石壁祖地祭祖大典暨第三届宁化石壁与客家世界学术研讨会，两岸交流得到加强。

　　围绕产业发展思路抓落实。加快产业结构调整，制定实施《振兴三明工业行动计划》、《三明市发展现代农业行动计划》、《三明市加快流通业发展行动计划》，实行一个产业、一个团队、一套政策、一个机制的运作模式，组织30个团队，着重抓好270个重点项目。着力突出、提升工业，围绕发展八大产业、打造六大基地，制定落实扶持重点工业企业的一系列措施，举办海峡西岸经济区（三明）投资项目推介会，强化与中央属、省属企业对接，拓展8个省级经济开发区，申报设立6个省级经济开发区，规模以上工业增加值303.91亿元，增长19.6%；工业经济效益综合指数201.3%，实现利税48亿元。推进技术创新，23个项目列入国家科技计划，90个项目列入国家、省技术创新和技改提升项目，新增14家国家高新技术企业，高新技术产业产值突破100亿元，占规模工业产值的比重从上年的5.3%上升到12%。深化质量振兴活动，4家企业获批筹建国家专业标准化委员会工作组。着力加强、做特农业，出台稳定粮食生产等产业扶持措施，支持龙头企业做大做强，推进产业基地建设，农林牧渔业总产值233.1亿元，增长6%；粮食产量113.26万吨，居全省第二位，宁化县被评为全国粮食生产先进县；林竹、果蔬产业产值超过50亿元；3家企业获省品牌农业企业金奖,设立3个全国农产品加工创业基地；新增8个省级农业标准化示范区，新增4件国家地理标志产品保护和40个绿色食品、无公害农产品，组织制（修）订3项国家标准和20项省地方标准。农民专业合作社达到858个,居全省首位。着力培育、搞活三产，加快建设海西金属材料制品市场、明成冷冻产品物流配送中心等专业市场，宁化闽赣边贸建材交易市场、新华都三明购物中心建成开业。新设立14家建筑业企业，建筑业产值达97亿元，增长43.8%。稳步推进普通商品住房建设，商品房销售面积193.45万平方米，增长143.4%。实施《三明市旅游发展总体规划》，在中央电视台投放三明旅游形象广告，泰宁申报世界自然遗产通过世界自然保护联盟专家考察评估，重点旅游项目完成年度投资目标，新增3家、新建11家四星级以上旅游饭店，游客接待量增长20.5%，旅游总收入增长22.6%。

　　围绕区域发展思路抓落实。持续壮大市区经济，向梅列区、三元区下放48项审批及管理权限，市财政筹资2.86亿元，推进产业、基础设施、城市片区开发。强化产业拓展，联手壮大金属深加工、机械与汽车、商贸物流等产业，实施三钢二炼钢技改、城市物流园区建设等重点项目，市区生产总值达到173.89亿元，占全市的23.7%；市本级地方财政收入9.77亿元，增加1.31亿元，增长15.4%。强化重点片区建设，徐碧新城中央商务区加快实施，五星级酒店、沃尔玛购物广场等项目动工，白沙旧城改造持续推进，台江大桥主体工程基本建成。支持“一轴两翼”发展，编制《加快三明中心城市发展行动计划》，共建一批基础设施和公共事业项目，抓好市、县（区）联办园区，实现产值增长40.9%，税收增长35.5%。引导各县（市）发展特色经济，形成生物医药、优质烟叶、苗木花卉等一批海西特色产业,东南一翼的地区生产总值和财政收入分别增长13.1%、8%,西北一翼的地区生产总值和财政收入分别增长13.9%、19.1%。实施惠农工程和新村镇试点示范工程，抓好十件实事，新农村建设取得实效。加快苏区、老区发展，争取政策取得突破，苏区、老区产业实力、基础设施建设、民生保障得到加强。加大扶贫开发力度，省、市级扶贫重点村争取资金3.2亿元；投入7690万元,改善库区移民生产生活条件。

　　围绕改革开放抓落实。创新体制机制，持续深化集体林权制度及其配套改革，推进林木采伐管理制度改革，新增林权抵押贷款4.48亿元。开展农村土地承包经营权流转，新增土地流转面积8万亩。启动市直行政事业单位国有资产管理体制改革。稳步实施成品油价格和税费改革，落实取消政府还贷二级公路收费改革政策，开展医药卫生体制改革。持续扩大开放，强化项目对接，第七届“6·18”海峡项目成果交易会签约合同项目数和投资额保持全省前列。组织参加“9·8”等重点招商活动，赴长三角、珠三角等地开展经贸洽谈，支持企业扩大出口，国家、省、市扶持资金达到3000万元，实现出口退税4.65亿元，外贸出口从去年8月起实现增长。设立大田、尤溪沿海产业转移集中区，建设宁化、建宁、泰宁闽赣边界产业、物流园区，引进区外资金86.75亿元。深化明港澳合作，加强与海外华侨及国际友城联系，海关、检验检疫、外汇管理、贸易促进等工作取得新成效。壮大非公有制经济，制定《关于进一步鼓励和扩大民间投资的实施意见》，开展服务企业年活动，非公有制经济产值增长22.5%、税收增长16.1%，新增65家产值超亿元的非公有制企业、68家纳税超百万元的企业，引进4家总部经济企业落户市区。

　　（三）责任总体到位

　　落实环境保护和生态建设责任。实施30个重点节能项目、54个资源综合利用项目，淘汰落后水泥产能211万吨，新增新型干法水泥产能600万吨。实施73个减排项目，重点强化流域水环境综合整治，完成污水管网建设改造107.67公里，建成6座污水处理厂和3座垃圾无害化处理场，市区生活污水处理率达到80%以上；拆除禁养区内养殖场178家。饮用水源水质达标率100%，市区第二水源项目加快推进，市区空气优良天数占总天数的94.2%。加强生态建设，18个乡镇被授予福建省环境优美乡镇，3个村被授予福建省生态村。实行最严格的耕地保护制度，连续11年实现耕地占补平衡。

　　落实社会事业发展责任。强化素质教育，宁化县“双高普九”达标，初中毕业生升学率达94.5%，高考本科上线率居全省前列；组建6个职教集团、6个职教中心，新增3个省级重点专业和1个省级示范性实训基地、1个省级技能型紧缺人才培养基地；三明学院等高校教学水平继续提升。引进287名高层次人才和紧缺急需专业本科毕业生，实施22个国（境）外智力项目。建成海西（闽中）科技馆、数字科普馆，设立院士工作站，扶持博士后工作站发展。健全公共卫生服务体系，提高重大疾病防控能力，加强医疗质量安全管理，完善医疗纠纷调解机制，有效防控甲型H1N1流感。发展文化事业与产业，清流赖坊被评为中国历史文化名村，8个乡镇入选中国民间文化艺术之乡，永安闽台文化创意产业园列入省重点扶持计划。文学艺术、新闻出版、广播电视、社会科学不断进步。低生育水平保持稳定，出生人口性别比治理取得成效，将乐县被评为国家级、清流县和大田县被评为省级计划生育优质服务先进单位。开展全民健身运动，提升竞技体育水平，永安市被评为全国实施农民体育健身工程先进县市。统计服务质量不断提高，物价、民族宗教、水文、气象、地震和地方志工作取得新进展，妇女、儿童、残疾人和未成年人工作得到加强。

　　落实促进就业责任。做好高校毕业生、失业人员、农村富余劳动力、返乡农民工、退役军人等的创业、就业工作，新增城镇就业2.47万人，转移农村劳动力4.97万人。健全社会保障体系，完成省政府下达的养老、失业、医疗、工伤和生育保险任务，城镇居民医疗保险参保进度居全省前列，大学生和中小学生参加医保工作有序实施，新型农村社会养老保险试点和社区居民养老服务试点扎实开展，被征地农民社会保障工作稳步推进。企业退休人员养老金人均月增加127元，城市、农村低保人均月补助水平分别提高11元和5.3元，优抚对象待遇水平提高。

　　落实维护社会稳定责任。扎实推进“平安三明”建设，加强城乡社区警务和人民调解工作，有效排查化解矛盾纠纷，群众对社会治安满意率达到95.5%，连续两届获得全国社会治安综合治理优秀地市称号。增强应对自然灾害和突发事件的能力，开展“安全生产年”、“责任落实年”活动，安全生产保持平稳态势。推进村（居）民自治，全市社区（居委会）全部完成换届，村委会换届完成99.8%。积极争创全国“五五”普法工作先进市，增强全民法制意识。深入开展群众性精神文明创建活动，第四次获得全国创建文明城市工作先进城市称号,沙县被评为全国文明县城。深化双拥共建,重视人民防空、国防后备力量建设,驻明部队和民兵预备役力量在经济建设、抢险救灾、维护稳定等方面发挥了重要作用。

　　（四）承诺总体兑现

　　强化重点工作落实。对重点工作、重要项目、民生实事进行责任分解，特别是将市十一届人大四次会议通过的《政府工作报告》，细化成118项重点工作，全力组织实施，在这次会上书面报告落实情况。各县（市、区）政府也建立了重点工作、重要项目责任分解、督查跟进、落实报告制度。同时，认真办理221件人大代表建议和292件政协提案，人大代表、政协委员提出的合理化建议得到充分采纳和较好落实。

　　强化民生项目实施。抓好省、市为民办实事项目，完成年度目标任务。同时，惠民项目取得新进展，教育方面，完成58个农村中小学寄宿制学校宿舍、食堂建设工程，国家助学金及减免学费等政策落实到位。医疗卫生和人口计生方面，完成乡镇卫生院、社区卫生服务中心和计划生育服务站（所）年度建设与改造任务；对边远建制村卫生所实行专项资金补助；提高农村部分计划生育家庭奖励扶助标准；完成贫困白内障患者复明手术、残疾人康复工程年度任务。文化建设方面，新建、改扩建27个乡镇综合文化站，建成190个“农家书屋”，完成有线数字电视整体转换4.43万户，农民体育健身工程完成年度任务。城市公共设施方面，完成社区基础设施年度改造任务，贵溪洋综合停车场进展顺利，下洋体育健身中心加快建设；老龄、社会福利和慈善事业持续发展，市老年活动中心、老年公寓、社会福利中心等项目加快实施。社会保障方面，政策性农业保险试点工作扎实推进，自然灾害救济救助公众责任保险实施；17所农村敬老院新建、改扩建任务基本完成；建成754套廉租住房、1020套经济适用住房。强农惠农方面，24个乡镇、330个村通过省级农村家园清洁行动验收；完成农村户用沼气池建设、农村危房改造、“造福工程”年度任务。援建灾区方面，完成7个对口支援彭州市红岩镇项目建设。食品药品安全方面，治理“餐桌污染”、建设“食品放心工程”取得成效，农产品质量安全检验检测体系不断完善。

　　强化开明、服务、法治、廉洁政府建设。提升行政效率，建立项目“快报”、“快批”、“快建”服务机制，对48个市直部门实行审批在线监察，形成市县乡联网的网上审批服务体系。完善政府绩效管理制度和行政问责制度，落实“四个不让”行为准则，开展“五比”竞赛活动，解决历史遗留的企业土地证、房产证办证问题，投资软环境满意度居全省第二位。创新运作机制，组建三明城市建设投资公司等7个平台，完善市重大项目工作组运作模式，提高重大项目前期经费，项目运作取得实效。健全现场调研、当场协调、限期解决的工作机制，逐项突破发展难题。加强依法行政，推进政务公开和政府信息公开，推行行政执法责任制，做好行政复议工作，加强行政监察、审计监督，规范行政行为。认真执行人大及其常委会决议、决定，自觉接受人大及其常委会的法律、工作监督和政协的民主监督，接受司法监督、社会监督。密切与各民主党派、工商联、无党派人士的联系与协商，支持工会、共青团、妇联等群团组织开展工作。推进廉政建设，落实党风廉政建设责任制，加强惩治和预防腐败体系建设。坚持勤俭节约，减少一般性行政开支。进一步规范行政权力运行，压缩行政自由裁量权空间，深化公共资源市场化配置，规范市场中介组织，纠正损害群众利益的不正之风。

　　各位代表，过去一年的成绩来之不易，是在省委、省政府和市委正确领导下，在历届政府打下的良好基础上，经过全市上下奋力拼搏取得的。在此，我谨代表市人民政府，向辛勤工作在各条战线的全市人民，向给予政府大力支持与有效监督的人大代表、政协委员、各民主党派、工商联、各人民团体、无党派人士、离退休老同志、社会各界人士和全体纳税人，向为三明发展做出积极贡献的中央和省属驻明单位、驻明部队、武警官兵、公安干警，向关心支持三明发展的港澳台同胞、海外侨胞、国际友人，致以衷心感谢和崇高敬意！

　　我们清醒地看到，在目标完成方面，虽然外贸出口增幅居全省第二位、社会消费品零售总额增幅居全省第三位，但这两项指标增幅未能完成预期目标；由于规划调整、道路走向变化等原因，城市通道建设等工作没有实现年度力争目标；三次产业比重、城镇化率等指标完成情况与“十一五”规划序时目标有较大差距。在产业发展方面，产业结构调整力度不够大，第三产业发展成效不突出；生产性大项目、好项目不多的问题没有突破，科技创新和人才支撑能力较弱。在城乡统筹方面，中心城市集聚作用不明显，部分县经济总量较小；“三农”工作成效不够突出，农村基础设施薄弱，农业产业化水平不高，农民持续增收难度增大。在公共服务和社会管理方面，防灾减灾体系还需完善，节能减排压力较大，影响安全生产、社会稳定的因素依然存在；就业结构性矛盾解决不够好，文化教育、医疗卫生、住房等民生保障与人民群众需求有较大差距。在政府自身建设方面，一些单位和地方的服务意识不强，行政效率不高，工作责任不够落实，服务环境不够优化。对此，我们将高度重视，采取更加有力措施，切实加以解决。

　　二、2010年工作安排

　　今年是我市实施“十一五”规划的最后一年，也是全面落实海西决策部署的关键一年。从总体上看，今年经济发展面临的积极变化和不利影响同时显现，我们要保持清醒头脑，抢抓发展机遇，做到谋划高起点，运作大手笔，力求大变化，实现好中求快、又好又快。总体要求是：全面贯彻党的十七大和十七届三中、四中全会与中央经济工作会议精神，以邓小平理论和“三个代表”重要思想为指导，深入贯彻落实科学发展观，按照省委八届六次、七次全会和市委七届六次、七次全会以及省、市经济工作会议的各项部署，进一步突出发展、注重运作、凝聚合力、弘扬正气，加快落实“一轴两翼”的区域发展思路和三次产业协调推进的产业发展思路，着力融入海西建设全局，着力扩大需求促进增长，着力转变发展方式，着力统筹城乡发展，着力深化改革开放，着力改善民生发展社会事业，推动全市经济社会更好更快发展，努力把三明建设成为连接沿海、辐射内陆、联动周边的海峡西岸经济区重要增长极。

　　经济社会发展的主要预期目标是：全市生产总值增长12.5%，地方级财政一般预算收入增长12%，全社会固定资产投资增长25%，外贸出口增长8%，按可比口径实际利用外商直接投资增长10%，社会消费品零售总额增长17%，城镇居民人均可支配收入实际增长8%，农民人均纯收入实际增长6%，居民消费价格总水平涨幅控制在3%左右，城镇登记失业率控制在4%以内，人口自然增长率控制在7‰以内，单位生产总值能耗降低4.2%，完成“十一五”规划确定的二氧化硫和化学需氧量减排任务。围绕上述目标要求，着重从以下五个方面开展工作：

　　（一）乘势而上促增长。继续用足用好用活扩大内需政策，发挥投资、消费的拉动作用，巩固经济回升向好基础，推动经济较快发展。

　　投资是拉动三明经济增长的重要动力，我们要持续加快基础设施建设，交通方面，完成公路投资50亿元以上，上半年永武高速公路三明段建成通车，年内基本完成永宁高速公路路基工程，建泰高速公路路基工程全线开工，中心城市快速通道和三明至明溪联络线动工，加快推进尤溪至莆田兴化湾、厦门至沙县、永安至漳州高速公路三明段前期工作；开工建设三明国家公路运输枢纽和一批县、乡（镇）客货运站；建设、改造省道496公里，完成国、省干线路面改善工程省定任务。向莆铁路三明段完成投资35亿元，力争南三龙和浦建龙梅铁路三明段控制性工程动工，长永泉铁路预可研方案竞选获批。下半年动工建设三明沙县机场。电网建设方面，计划投资8亿元，开工建设一批重点输变电工程和县域电网改造提升项目，加快500千伏永安输变电项目前期工作。农林基础设施方面，完成农田基础建设投资6.2亿元以上，土地整理（治理）15万亩以上，新建竹山道路1000公里。健全防灾减灾体系，抓好农村民生水利工程，健全动物疫病和农业、林业有害生物防控体系。持续加快重点建设，安排163个市级重点建设项目，力争完成投资162亿元，实现60个项目投产或部分投产，50个以上预备项目动工建设。抓紧实施16个社会事业重点项目，完成投资10亿元以上。持续加快生产性项目建设，实施列入省重点产业调整振兴方案的94个项目，并继续跟进、争取。同时，围绕工业、现代农业、流通业行动计划，抓紧实施270个重点项目，突出抓好68个投资亿元以上工业项目。全力完成“十一五”规划任务，精心编制“十二五”规划，着力策划一批重大项目。

　　把扩大消费作为促增长的重要举措，提高消费能力，通过落实惠农政策、发展农村二三产业等途径，促进农民增收；提高企业退休人员基本养老金、部分优抚对象待遇水平；落实中小学校、公共卫生与基层医疗卫生事业单位绩效工资政策；推动工资集体协商和工资支付保障制度建设，落实最低工资制度和对低收入群体的转移支付制度。拓展消费空间，落实鼓励家电、汽车、摩托车消费的举措，兑现农机具购置补贴政策，培育新型消费业态。推进供销社改革和发展，加强农村流通体系建设，健全经营服务和购销网络，改造提升乡镇集贸市场。优化消费环境，加强重要商品、居民生活必需品的价格监测和调控保障。持续发展房地产业，落实稳定房地产市场、解决低收入家庭住房困难问题的工作责任制，加快中低价位、中小套型普通商品住房建设，加大旧城区、棚户区、农村危房改造力度，推进保障性安居工程，扩大廉租住房保障范围。合理引导住房消费，加强商品房销售管理，促进房地产市场健康发展。

　　把握货币政策导向，加大信贷投入，健全政府支持金融、金融刺激经济的机制，加大对“三农”、重点产业、重点企业和中小企业等方面的信贷支持，力争新增贷款140亿元。抓紧组建三明市区农村商业银行和小额贷款公司，争取区外股份制银行在我市设立分支机构，在所有乡镇设立金融网点和自动设备终端。加强社会信用体系建设，营造良好金融生态环境。延伸融资平台，深化与政策性金融机构的合作，以市城市建设投资公司等平台为重点加强资源、资产、资本运作，推进与兴业证券、华兴创投等公司的战略合作。加强政府信用贷款投资管理，健全融资平台激励约束机制和风险防范机制。推进企业直接融资，引导企业发行企业债券、短期融资券和中期票据，鼓励发展租赁、信托、典当等行业和机构。推进2家以上企业上市，引导上市企业做好配股和增发工作，实现三农公司股票恢复上市。

　　（二）抢抓机遇调结构。优化经济结构是转变经济发展方式的基本要求，必须把握有利时机，在壮大总量中促进经济结构调整，在经济结构调整中提升发展水平，增强区域经济实力。

　　有规模才有调整的基础，必须做大总量、延伸产业。着力培育壮大百亿产业，围绕发展先进制造业，实行“四个一”的运作模式，安排5000万元工业发展专项资金，深入实施《振兴三明工业行动计划》，重点做大做强4个产值超百亿产业，推动矿产品深加工、化工产业进入百亿产业行列。扶持省一建公司做大做强，推动20家年产值亿元以上的建筑业企业加快发展，力争建筑业产值突破120亿元。做强做优农业特色产业，落实《三明市发展现代农业行动计划》，稳定粮食播种面积，推广关键增产技术，确保粮食生产总水平基本稳定；加快发展特色农业产业，率先做大做强林竹、果蔬、畜牧水产、油茶、茶叶等5个重点产业；依托健盛食品、沈郎食用油等龙头企业，重点建设10个特色农业基地。加快发展现代服务业，落实《三明市加快流通业发展行动计划》，大力发展现代物流业，抓紧建设海西金属材料制品市场、三明城市物流园区，加快推进三明“无水港”、华油仓储物流中心等项目，抓好金属材料交易等物流信息平台建设。拓展、提升交通运输业，积极发展会展经济，支持发展金融、保险业，鼓励发展科技服务、法律咨询等服务业，壮大社区服务业，培育信息服务业和外包服务业。持续拓展旅游产业，推广“泰宁路径”旅游发展模式，抓紧组建旅游集团公司，全力争取“中国丹霞”泰宁列入世界自然遗产名录，支持建设海峡旅游（泰宁）产业园等8个重点旅游项目，力争新增5家四星级以上旅游饭店，规划建设三明旅游集散服务中心。依托中央电视台等主流媒体，加大旅游宣传推介力度，办好重点旅游节庆，着力拓展高速公路沿线客源市场，深化明台旅游合作，力争游客量突破1000万人次。

　　发展战略性新兴产业是结构调整的方向，必须发挥优势、持续拓展。加快发展生物医药及生物产业，加强与国内外高校、科研机构合作，引进国内知名制药企业，着力扶持5家重点企业，建设7个重点种植基地，打造全国中药材种植示范区。加快推进新能源产业，抓紧三明示范快堆核电项目前期工作，实施大唐国际生物质能发电一期工程，建设建宁生物质能源基地及系列加工项目。加快培育新材料产业，实施不锈钢复合板项目，抓好无机粉体造纸项目前期工作，推进稀土产业发展。加快提升节能环保产业，开发节能环保装备，做大垃圾压实机等环保装备制造业，推进双能源汽车、抽水蓄能发电等项目前期工作。

　　企业是结构调整的主体，必须突出重点、壮大龙头。扶持工业骨干企业，加大对38家成长型工业企业的扶持力度，力争新增4家产值超10亿元企业。支持在明中央属、省属企业新上项目、资产重组，加强与国内外大企业特别是央企的项目对接。壮大农业产业化龙头企业，研究制定农业产业化发展的扶持办法，重点扶持永安林业、三和食品等10家产值超5亿元的龙头企业，力争新增5家国家级扶贫龙头企业与省级龙头企业。培育流通业重点企业，支持商业、物资等流通企业加快发展，推动海鑫等物流企业做大做强，促进明成冷冻产品等专业市场加速拓展，新增10家年销售额亿元以上商贸物流企业。落实扶持中小企业加快发展的政策措施，创造企业成长的良好条件。

　　转变粗放发展方式是经济结构调整的重要任务，必须提升内涵、集约发展。走技术创新的路子，持续推进创新型城市建设，力争55个以上项目列入国家、省级科技计划，实施50个技术创新和高新技术产业化重点项目，力争高新技术产业产值突破150亿元。建成金属深加工、载重汽车等5个重点特色产业技术联盟，建设企业技术中心、博士后工作站等研发平台，动工建设省煤气化工程技术研究中心。持续对接、落实“6·18”项目，力争签约项目数、总投资额、动工率继续走在全省前列。走品牌带动的路子，支持、奖励企业争创品牌，新增2个中国名牌产品，继续争创中国驰名商标；新认证20个有机食品、绿色食品、无公害农产品，新增2件以上国家地理标志产品保护；鼓励企业参与制（修）订国家、地方和行业标准。走节能减排的路子，大力发展低碳经济，严格落实节能减排工作责任制，淘汰落后水泥产能100万吨以上，实施30个年节标煤千吨以上项目，深化清洁生产试点城市建设，推动循环经济示范试点建设，开展全国再生资源回收体系建设试点城市工作；严把新上项目产业政策、资源消耗和环境保护关，实施40个减排项目，推进流域水环境综合整治，治理畜禽养殖和工矿企业污染，推进污水、垃圾处理项目加快建设、规范运营。加快生态公益林建设，继续推进市区第二水源项目，强化饮用水源地保护；控制农村面源污染，继续开展农村家园清洁行动，支持生态县创建工作，力争创建一批国家级、省级环境优美乡镇和生态村。运用土地价格调节机制，引导节约、集约用地。

　　（三）集聚合力抓拓展。进一步调动各方积极性，促进要素集聚，拓展发展空间，构筑发展平台，加快城乡建设，提升城镇化水平，实现城乡统筹发展。

　　以中心城市发展带动区域实力增强。落实《加快三明中心城市发展行动计划》和扶持市区经济发展措施，继续做大做强市区经济，推进市县一体化和城市联盟，进一步壮大主轴，增强中心城市的辐射力、带动力。抓紧规划衔接，完成三明市区、永安、沙县中心城市战略规划，合理布局中心城市重点区域、重点项目、重点板块，协调中心城市建筑风格与色彩，改善城市重要节点景观。加快片区开发，启动贵溪洋新区开发，加快徐碧新城建设进度，促进下洋中区拓展延伸，推进台江片区规划建设，重点抓好五星级酒店等21个项目，动工建设三明体育场馆等一批标志性建筑。支持永安、沙县加大片区开发力度，建设精品工程。推动产业集聚，依托共建园区和工业集中区，共同打造金属深加工、机械及汽车制造、林产工业、纺织印染、生物医药及生物产业、现代物流等产业集群，力争“主轴”地区生产总值增幅高于全市平均水平；制造业产值占全市的60%以上，增长20%以上。完善城市配套，推进中心城市道路、市政设施的规划、建设，完成江滨路、江滨南路改造，动工建设西江滨路改造工程；调整中小学布局，扩大优质高中办学规模，完善医疗服务设施，繁荣城市特色文化；深化户籍管理制度改革，鼓励外地人员到三明投资创业、生活居住，不断集聚中心城市人气。

　　以县城建设带动县域经济发展。提升县城建设水平，总结推广泰宁县城建设经验，立足各县的特色和文化，确定县城建设的定位、风格和色彩。推动各县开发一批新区精品，有序实施旧城改造，提升层次和品位。有计划有步骤地解决农民工在城镇就业和生活问题，放宽中小城市特别是县城和中心镇落户条件。打造县域特色产业，按照立足优势、突出特色、参与分工、各自竞跑的要求，支持各县（市、区）培育各具特色的县域产业，力争每个县（市、区）都打造一个以上在全省有影响、有实力的特色产业，建设经济强县。

　　以中心镇建设带动城镇化水平提高。加快中心镇建设，按照“规划先行、功能齐备、设施完善、消费便利、保障一体”宜居城市综合体的建设要求，加快小城镇建设，着力推进高速公路互通口、快速铁路客（货）运站所在镇发展和环境整治，着重抓好17个重点中心镇建设。健全新型农村社会化服务体系，进一步夯实农业农村发展基础，推进农业标准化生产，促进农业“五新”进村入户。稳定“农业155”联动工作队伍，加快乡镇“三农”服务中心建设，健全基层农技服务体系，推进沙县、建宁国家基层农技推广体系改革与建设示范县工作，新增50个农民专业合作社。办好新农村建设实事，完善农村服务配套功能。扶持革命老区和欠发达地区发展，用好用足国家、省里支持苏区老区发展的政策，进一步加大扶持力度，推动产业、基础设施、社会事业发展；加大扶贫开发力度，支持少数民族乡村发展，抓好扶贫开发重点村建设，继续推进库区移民安置和后期扶持工作。

　　以园区建设带动要素集聚。加快海西三明台商投资区开发，推进吉口新兴产业园建设，重点发展资源深加工、生物医药及生物产业、装备制造等产业。抓紧完善海西三明现代物流产业开发区规划，推进向莆铁路三明北站等项目建设。拓展、提升现有8个省级经济开发区，力争获批4个以上省级经济开发区，加快建设8个特色产业园区，并从投资额、投资强度、园区产出、实现税收等方面，对园区经济进行分类考核。

　　（四）先行先试增活力。以改革增动力，以开放添活力，不失时机推进重要领域和关键环节改革，不遗余力扩大对外开放和区域合作，增创科学发展、加快发展新优势。

　　以深化林业改革为重点，创新体制机制。持续推进集体林权制度配套改革，加快永安全国林业改革与发展示范区建设，整合市、县国有林业资源、资产，探索林业资本化运作方式，开展政策性森林保险，鼓励林农建立林业专业合作社。积极推进城乡统筹改革试验，稳定和完善农村基本经营制度，健全农村土地管理制度，稳妥推进土地承包经营权流转，发展多种形式的适度规模经营。以推广“168”基层党建工作模式为抓手，创新农村工作机制。推进国有资产经营管理体制改革，以市国投公司等企业为主，推动重点产业和公共事业发展，促进国有资产保值增值。深化部门预算改革，优化财政支出结构，严格控制一般性支出，继续压缩行政事业单位公用经费。完成市、县（区）政府机构改革，基本完成乡镇机构改革，推进事业单位分类改革。

　　以推动明台合作为重点，拓展开放领域。打造两岸交流合作平台，加快海峡两岸现代林业合作实验区和清流台湾农民创业园建设，推进海峡两岸林业博览园建设，办好第六届海峡两岸林业博览会，赴台开展小吃文化旅游及项目推介活动。推进客家文化生态保护实验区建设，组织参加海峡论坛，申办第25届世界客属恳亲大会，实施永安闽台文化创意产业园等项目，推动两岸文化、教育、卫生等交流。打造对外经贸合作平台，着力培育15—20家生产型出口龙头企业，重点拓展东盟、中东、非洲等新兴市场，提高地产品出口比重。开展系列招商活动，依托“9·8”等平台，注重引进先进技术、管理经验和智力资源，引导外资投向产业集群、现代服务业等领域，支持现有外商投资企业增资扩股。加强与港澳地区、国外友城的沟通联系，做好海外华侨华人联络工作。打造区域合作平台，加快大田、尤溪沿海产业转移集中区建设，联手推进跨区域项目，推动宁化、建宁、泰宁闽赣边界产业、物流园区建设，打造边界产业带。

　　以壮大非公有制经济为重点，增强发展活力。落实鼓励和扩大民间投资的政策，进一步放宽非公有制企业投资领域和市场准入。着力解决企业用工、融资、用地等难题，扶持成长型非公有制企业，力争新增产值超亿元企业20家、税收超百万元企业50家。发展商会经济，实施“回归工程”，支持明商回乡投资兴业。鼓励非公有制企业体制创新、技术创新、管理创新，加强企业家队伍建设，保护企业合法权益。

　　（五）实干实效保民生。把改善民生、发展社会事业作为扩大内需、调整经济结构的重点，着力完善公共服务，着力加强社会管理，着力解决事关群众切身利益的问题，促进社会和谐稳定。

　　强化民生民本。落实更加积极的就业政策，深化国家级创业型城市创建工作，力争通过国家验收；加快就业平台建设，加强职业技能培训和农村劳动力转移培训，健全就业援助体系，促进高校毕业生、城镇困难人员、退役军人等重点人群就业，保持城镇“零就业家庭”动态为零，力争全年新增城镇就业2.2万人。扩大社会保险覆盖面，实行城镇职工医疗、工伤、生育保险市级统筹，落实养老保险异地转移接续制度，在大田、尤溪开展新农合门诊补偿试点，提高城乡低保标准。健全灾害救助机制，提高农房统保、自然灾害责任保险等水平。发展社会福利事业，维护和保障妇女儿童合法权益，加强老龄工作，积极发展慈善事业。

　　完善公共服务体系。优先发展教育，推进素质教育，加快城乡中小学标准化建设，扩大优质教育资源，加强教师队伍建设，促进义务教育均衡发展。组建高级技工学校，推进职业教育集团、职教中心和公共实训基地建设,加快高等院校基础能力建设，重视早期教育、幼儿教育，扶持特殊教育，发展继续教育和老年教育，支持和规范民办教育，关心外出务工人员子女的学习、生活。加强人才队伍建设，完善人才服务体系，健全人才培养、引进、使用、激励机制。抓好引智基地建设，加快引进高层次和紧缺急需专业人才。提升公共卫生服务水平，推进医药卫生体制改革，年内60%的基层卫生服务机构推行基本药物制度。加强甲型H1N1流感等重大疾病防控和卫生监督，坚持中西医并重，发展妇幼保健事业。围绕稳定低生育水平，加强流动人口管理和服务，综合治理出生人口性别比偏高问题，建立免费孕前优生健康检测制度，提高出生人口素质。推动文化事业与产业发展，重视文物工作和非物质文化遗产的传承与保护，规划建设市图书馆、艺术馆等设施，规范网吧管理。扶持发展动漫、印刷发行等文化产业，培育文化龙头企业，加强文化市场建设。启动各县（市）数字电视整体转换，提高服务质量。繁荣社会科学，发展新闻出版、文学艺术等事业。保护知识产权，办好院士工作站，普及推广科学知识。继续加强统计工作，开展第六次全国人口普查，促进民族宗教、档案、史志、水文、气象、防震等工作全面进步。提高全民健身运动和竞技体育水平，争取在第十四届省运会上取得好成绩。

　　落实惠民利民项目。集中新增财力办好为民办实事项目，教育卫生方面，实施中小学校舍安全工程；巩固发展新型农村合作医疗和城镇居民基本医疗保险;推进城乡公共卫生服务工程，加强城市社区卫生服务，着手建立全民健康信息平台；实施乡镇卫生院加强建设规划、医师帮扶基层医疗卫生机构、乡村医生培训工程，继续补助边远建制村卫生所。文体和公共设施方面，建设10所乡镇综合文化站，实施农村电影放映工程和“农家书屋”工程；向公众免费开放仙人谷国家森林公园；实施农民体育健身工程，推动市区重点体育设施建设；完善城市社区基础设施，市区新增停车泊位4000个以上，建成市老年活动中心；实施明溪县黄沙坑水库等县城供水工程。社会保障方面，开展大田县国家级新型农村社会养老保险试点；建设10所农村敬老院和1个县级社会福利中心项目、建立40个社区居家养老服务中心（站）；加快保障性住房建设，新开工廉租住房1500套，建成经济适用住房2000套；实施特殊教育学校提升工程、“光明行动”，建设残疾人“福乐家园”，资助残疾人居家托养，实施残疾儿童康复项目，争创全国残疾人工作示范城市。强农惠农方面，实施水源保护、饮水安全、环境整治工程；继续抓好农村沼气工程、农村改厕项目；提高森林生态效益补偿标准；继续实施“万村千乡”市场工程；建设农村社区综合维修服务体系；开展农村实用技术远程培训；继续推进农村公路路网工程，提高农村公路养护补助标准；抓好水库除险加固工程；实施“造福工程”；完善政策性农业保险试点工作；实施城乡通信信息化提升工程。和谐平安方面，围绕争创全国综治“长安杯”，深化新一轮“平安三明”建设；治理“餐桌污染”，建设“食品放心工程”；继续帮助驻明部队建设生产生活项目。全面完成省下达我市的彭州市地震灾区援建任务。

　　加强社会管理。推进社会主义核心价值体系建设，持续抓好精神文明创建和未成年人思想道德工作，以“满意在三明”为主题，以争创全国文明城市、全国未成年人思想道德建设先进城市为抓手，不断提高公民文明素质和社会文明程度，促进城市管理规范化、制度化、法制化。扎实推进依法治市，争创全国“五五”普法工作先进市，抓好社会矛盾化解、社会管理创新、公正廉洁执法三项重点，推进公安信息化、执法规范化、构建和谐警民关系三项建设，完善社会治安防控体系，遏制违法犯罪。强化信访督查督办，依法依规处理信访问题，预防和妥善处置群体性事件。强化安全生产“一岗双责”和企业主体责任，完善安全生产监管、防控、应急管理体系，深入开展隐患排查治理，遏制重特大安全事故发生。注重推进村（居）民自治工作，扩大基层群众自治范围。加强与各民主党派、工商联、无党派人士的联系与协商，支持工会、共青团、妇联等人民团体依照法律和各自章程开展工作。支持驻明部队和武警部队训练，加强军地沟通，抓好民兵预备役、国防动员、人民防空、双拥共建工作，妥善安置转业、退伍军人，巩固军政军民团结。

　　三、切实加强政府自身建设

　　建设开明政府，突出增强创新力。坚持先行先试，进一步解放思想，始终保持积极向上的朝气、开拓创新的锐气、攻坚克难的勇气，把握国家支持海西建设、发展方式转变、三明区位改善等机遇，不断创新思路、创新举措。善于谋划策划，更加主动研究政策、延伸政策、落实政策，锲而不舍争取支持；更加主动策划项目，着力在产业政策中找项目，在产业链填平补齐中找项目，在产学研合作、区域协作中找项目；更加注重科学民主决策，深化政务公开和政府信息公开，落实《三明市人民政府重大事项决策程序规定》，建立健全公众参与、专家论证和政府决定相结合的行政决策机制，实行决策责任追究制度。着力重点突破，对重点工作、重要项目、民生实事，继续进行责任分解，在市“两会”上报告落实情况。对发展难题不回避、不掩饰、不敷衍，精心组织，强力运作，逐项突破。

　　建设服务政府，突出增强执行力。提高团队运作实效，调整、配强重大项目团队，增加项目前期经费，完善考评机制，确保重点工作、重大项目实现突破。促进行政审批提速，深入推进行政审批制度改革，强化各级行政服务中心建设，推进省、市、县网上审批联动联办，完善视频监控系统和审批监察系统。深化机关效能建设，完善政府绩效管理，加强公务员队伍建设，落实“四个不让”行为准则，凡事不能简单地说不好办、办不了，必须主动换位思考，真正替对方着想；凡事必须想在前、帮在前、做在前，今天能办的不拖到明天，自己能办的不推给他人。

　　建设法治政府，突出增强公信力。强化法治意识，认真对人大及其常委会负责并报告工作，办好人大代表建议和政协提案，自觉接受人大及其常委会法律监督和工作监督、政协民主监督及舆论监督、社会监督。深入落实国务院依法行政纲要、加强市县政府依法行政的决定和省政府实施意见，把政府工作纳入法制轨道。推进依法行政，加强行政复议工作，依法维护公民、法人和其他组织的合法权益，减少对微观经济运行的干预，切实落实减轻企业负担的一系列政策措施，严禁向企业乱检查、乱评比、乱收费。规范行政执法，开展相对集中行政处罚权和综合执法试点，加强依法行政综合监察，规范行政许可、行政征收、行政强制自由裁量权，完善行政执法责任追究机制，提高行政执法效率和水平。

　　建设廉洁政府，突出增强约束力。注重自律，深入开展反腐倡廉教育，筑牢思想道德防线，严格执行党风廉政建设责任制，落实廉洁自律各项规定，厉行节约，秉公用权，干净干事。健全制度，落实公共资源市场化配置制度，完善房地产开发、城乡规划管理、国有土地使用权“招拍挂”出让等制度，严格执行政府采购、产权交易制度，积极推行政府投资项目代建制、公示制。强化监督，加强对政府投资决策和资金安排使用的监管，规范工程建设、林业、土地、矿产资源开发等交易，严肃查办违纪违法案件，努力做到为民、务实、清廉。

　　各位代表！海西建设进入新阶段，三明发展面临新机遇。让我们紧密团结在以胡锦涛同志为总书记的党中央周围，高举中国特色社会主义伟大旗帜，以邓小平理论和“三个代表”重要思想为指导，深入贯彻落实科学发展观，在市委的正确领导下，团结和依靠全市人民，把握机遇、乘势而上，开拓创新、奋力拼搏，为推进海峡西岸经济区建设，实现三明又好又快发展而努力奋斗！
